# Hepatic spheroids derived from human induced pluripotent stem cells in bio-artificial liver rescue porcine acute liver failure

**DOI:** 10.1038/s41422-019-0261-5

**Published:** 2019-12-11

**Authors:** Sitong Chen, Jinglin Wang, Haozhen Ren, Yinan Liu, Chengang Xiang, Cheng Li, Shichun Lu, Yan Shi, Hongkui Deng, Xiaolei Shi

**Affiliations:** 1 Department of Cell Biology, School of Basic Medical Sciences, State Key Laboratory of Natural and Biomimetic Drugs, Peking University Health Science Center and the MOE Key Laboratory of Cell Proliferation and Differentiation, College of Life Sciences, Peking-Tsinghua Center for Life Sciences, Peking University, Beijing, 100191 China; 2 Department of Hepatobiliary Surgery, the Affiliated Drum Tower Hospital of Nanjing University Medical School, Hepatobiliary Institute of Nanjing University, Nanjing, Jiangsu China; 3 Center for Bioinformatics, Peking University, Beijing, 100871 China; 4 Department of Hepatobiliary Surgery, Chinese PLA General Hospital, Beijing, 100853 China

**Keywords:** Stem-cell differentiation, Induced pluripotent stem cells

Dear Editor,

Acute liver failure (ALF) is a complicated disorder showing a nearly 80% mortality rate, and there is currently no effective medical solutions for ALF except liver transplantation.^1^ Bio-artificial liver (BAL) system is a device that consists of a bioreactor filled with hepatocytes, which could potentially rescue ALF patients by providing partial liver function until a suitable donor liver can be found or the native liver has self-regenerated.^2,3^

Currently, the lack of a stable and clinically applicable hepatocyte source has impeded the application of the BAL system for ALF treatment. The scarcity of human liver donors has made primary human hepatocytes (PHHs) an impractical cell source to meet the cell number requirement of the BAL system, which is in the order of magnitude of 10^9^.^4,5^ Porcine hepatocytes are unsuited for application in BAL devices because of potential risks including immunogenic response and xenozoonosis, whereas hepatoma cell lines are limited by their incomplete functions. Human induced hepatocytes (hiHeps) using lineage reprogramming have been demonstrated to rescue ALF in large animal models.^6^ However, the limited lifespan of hiHeps leaves much to be desired for a stable and continuous cell source.

One potential strategy of generating hepatocytes for application with the BAL system is the use of human induced pluripotent stem cell (hiPSC) lines, which could stably and perpetually self-renew in vitro, serving as an optimal cell source for the generation of functional cell types.^7^ Moreover, the elimination of genomic integration and background oncogenic transgene expression makes hiPSC-derived cells a safe and promising cell source for clinical applications.^8,9^ However, it remains unknown whether hiPSC-derived hepatocytes can be efficiently generated to fulfill the requirement of large quantities of cells for the BAL device, and applied in the BAL system for ALF treatment.

To establish a strategy to generate large quantities of hepatocytes from hiPSCs, we further optimized our previously reported protocol, inducing hiPSCs to differentiate into hepatic progenitor cells (hHPCs) (Fig. 1a, b; Supplementary information, Fig. S1a). The α-fetoprotein (AFP) and albumin (ALB) positive hepatic progenitor colonies could be enriched to high purity in vitro under a chemically defined medium (hHPC expansion medium) (Fig. 1d; Supplementary information, Fig. S1b, c), and expressed hepatic progenitor markers including AFP, KRT19, HNF1B and FOXA2 (Fig. 1c; Supplementary information, Fig. S2a). RT-qPCR and RNA-Seq analysis indicated that these hHPCs expressed hepatic progenitor-specific genes at similar levels to freshly isolated fetal human hepatocytes (FHHs), but distinctly from hiPSCs (Supplementary information, Fig. S2b, c). These hHPCs could also be successfully cryopreserved and recovered (Supplementary information, Fig. S1d, e).

**Fig. 1:**
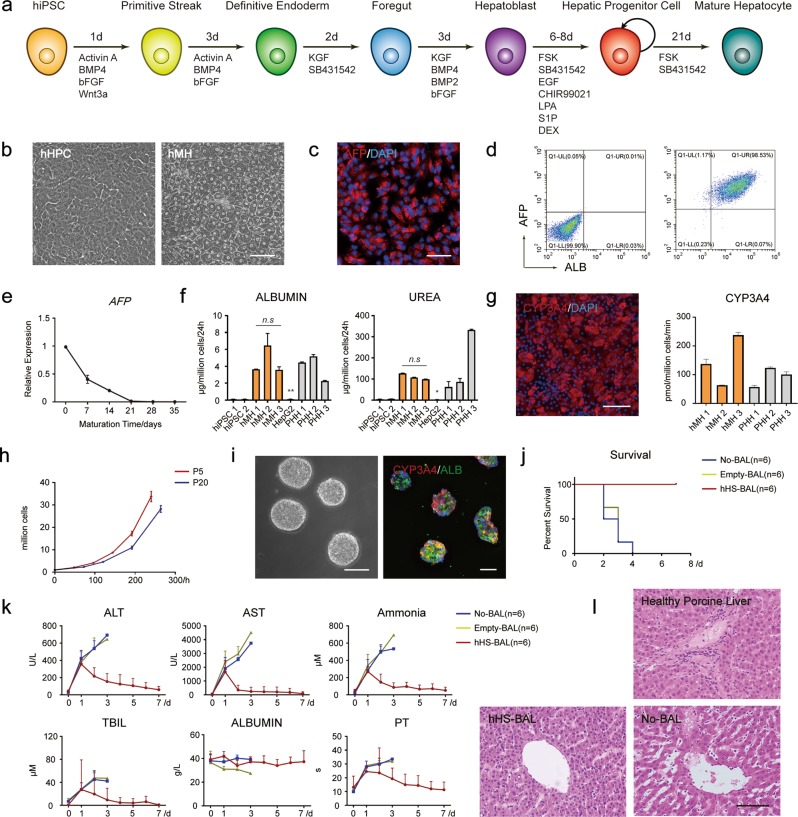
hiPSC-derived hepatocytes as an efficient cell source for the rescue of ALF disease in the BAL system. **a** Strategies of cell differentiation from iPSCs to hMHs. **b** The morphology of the hiPSC-derived hHPCs and hMHs. Scale bar, 100 µm. **c** Immunofluorescence of the expression of hepatic progenitor-specific marker AFP in hHPCs. Scale bar, 100 µm. **d** Differentiation efficiency of AFP- and ALB-positive hiPSC-derived hHPCs, as measured by flow cytometry analysis. **e** Quantitative comparison of the hepatic progenitor-specific gene AFP at mRNA level, which showed down-regulation during maturation in hHPC maturation medium from Day 0 to Day 21 and remained stable during continued culture from Day 21 to Day 35. *n* = 2. **f** Quantitative comparison of albumin secretion or urea synthesis among hiPSCs, hMHs, HepG2 cells and PHHs. hMHs 1–3 represent hMHs differentiated from three individual hiPSC lines, and PHHs 1–3 represent freshly isolated PHHs from three individual donors. *n* = 2. Data are presented as means ± SEM. *t*-test, *P *<* 0.05, **P *<* 0.01. **g** Expression of hepatic functional gene CYP3A4 measured by immunofluorescence and LC-MS using testosterone (Sigma) as substrate. Scale bar, 100 µm. *n* = 3. Data are presented as means ± SEM. *t*-test, *P* *>* 0.05. **h** Cell expansion curve of hiPSC-derived hHPCs in vitro from passage 5 to passage 9, and from passage 20 to passage 24. **i** Morphology and immunofluorescence analysis (ALB and CYP3A4) of functional hepatic spheroids in the low-speed stirring culture system in hHPC maturation medium for ~20 days. Scale bar, 100 µm. **j** Kaplan–Meier survival curve of ALF pigs in the hHS-BAL group, No-BAL group and Empty-BAL group (*n* = 6 for each group). **k** Detection of serum biochemical indices of ALF pigs in hHS-BAL group, No-BAL group and Empty-BAL group. Serum levels of ALT, AST, ammonia, TBIL and albumin, and prothrombin time (PT) were measured. **l** Hematoxylin and eosin (HE) staining of porcine liver samples in hHS-BAL group and No-BAL group. Healthy porcine liver was used as positive control. Liver samples in the No-BAL group were collected within 4 days post induction when the ALF pigs died. Liver samples in hHS-BAL group were collected on Day 7 after the ALF pigs were sacrificed. Scale bar, 100 µm

hHPCs could be further matured into functional hepatocytes when cultured in the hHPC maturation medium (Fig. 1a). Expression of hepatic progenitor marker AFP declined as maturation progressed within 20 days (Fig. 1e), following which hiPSC-derived mature hepatocytes (hMHs) showed a similar cell morphology to PHHs (Fig. 1b). The hMHs synthesized glycogen (Supplementary information, Fig. S3a), secreted albumin and urea (Fig. 1f; Supplementary information, Fig. S3b), detoxicated ammonia (Supplementary information, Fig. S4d), and expressed key CYP450 proteins (Fig. 1g; Supplementary information, Fig. S3c, d) and key hepatic transcription factors and functional genes (Supplementary information, Fig. S4a–c) at comparable levels to PHHs. RNA-Seq analysis of hMHs indicated that the expression patterns of important hepatic physiological functional genes were similar to those in PHHs (Supplementary information, Fig. S3e).

We further developed a two-step amplifying culture system to generate sufficient quantities of human hepatocytes (Supplementary information, Fig. S5a). Firstly, the hiPSC-derived hHPCs were expanded using a cascade amplification process in adherent culture in hHPC expansion medium for more than 20 passages in vitro, maintaining normal karyotype without alterations in HPC characteristics and proliferation rate (Fig. 1h; Supplementary information, Fig. S5b, c). Secondly, the hHPCs were transferred into a low-speed stirring culture system for floating culture in hHPC maturation medium to generate mature hepatic spheroids on a large scale. These mature hepatic spheroids expressed mature hepatic markers ALB and CYP3A4 (Fig. 1i), secreted albumin and urea (Supplementary information, Fig. S5d), and expressed important mature hepatic genes at comparable levels to PHHs (Supplementary information, Figs. S5e, 6).

To rescue ALF pigs, hiPSC-derived hepatic spheroids (hHSs; ~1 × 10^9^ cells) suspended in culture medium were assembled into our previously reported multilayer BAL device, where medium was exchanged with pig blood plasma^10^ (Supplementary information, Fig. S7a). Eighteen adult Bama miniature pigs were treated with D-gal to induce ALF. The pigs developed severe ALF symptoms in 24 h with significant increase in liver failure indices including alanine aminotransferase (ALT), aspartate aminotransferase (AST) and ammonia levels in the blood (Supplementary information, Fig. S7a, b and Tables S2–4). These animals were randomly assigned into three groups: a non-treated group (No-BAL, *n* = 6) in which no BAL was used, and two BAL-treated groups with BAL containing hHSs (hHS-BAL, *n* = 6), or no biomaterial (Empty-BAL, *n* = 6). Following 4-h treatment, the health status of the pigs in the hHS-BAL group showed apparent improvement 24 h after treatment (Supplementary information, Fig. S7c), and all six hHS-BAL-treated pigs successfully recovered and survived (Fig. 1j; Supplementary information, Table S1a). A significant down-regulation of ALT, AST, ammonia and total bilirubin (TBIL) levels in the blood could be detected in the ALF pigs of the hHS-BAL group (Fig. 1k; Supplementary information, Tables S2–4). In the other two groups, the pigs died within 4 days due to complications from ALF (Fig.1j; Supplementary information, Table S1b, c). The surviving pigs were sacrificed on day 7 to analyze the liver organ morphology, which revealed restored liver architecture and low immune response level; in contrast, untreated ALF pigs displayed signs of a liver injury state (Fig. 1l; Supplementary information, Fig. S7d). To evaluate long-term survival, we performed long-term observation of an additional hHS-BAL-treated ALF pig. Two months after BAL treatment, this pig showed excellent survival, without signs of ALF symptoms. The blood physiological indices of this pig including ALT, AST, ammonia and TBIL levels were restored to healthy levels in 7 days, and they were maintained subsequently (Supplementary information, Fig. S7e).

Here, we successfully established an efficient hiPSC-to-hHS differentiation strategy optimized for adaptation to the BAL system. The hHPCs could be scaled up, cryopreserved in vitro, and further matured to functional hepatic spheroids on a large scale with some critical physiological functions for ALF rescue comparable to PHHs, which could be more compatible for the BAL device in comparison to conventional hepatocyte culture systems.

Moreover, we demonstrated hiPSC-derived hepatocytes as an efficient cell source for the rescue of ALF disease in the BAL system, as 1 × 10^9^ hHSs were sufficient to rescue ALF pigs weighing on average 49.4 kg. This feature combined with the unlimited proliferation of hiPSCs allows for an off-the-shelf hepatocyte bank for the stable production of functional hepatocytes in large quantities, well adapted for application with the BAL system and future ALF therapy.

## Supplementary information

Supplementary information

## References

[1] Bernal W (2013). N. Engl. J. Med..

[2] Glorioso JM (2015). J. Hepatol..

[3] Chen HS (2019). Hepatology.

[4] Struecker B (2014). Nat. Rev. Gastroenterol. Hepatol..

[5] Robinton DA (2012). Nature.

[6] Shi XL (2016). Cell Res.

[7] Shi Y (2017). Nat. Rev. Drug Disco..

[8] Trounson A (2016). Nat. Rev. Mol. Cell Biol..

[9] Kimbrel EA (2015). Nat. Rev. Drug Disco..

[10] Shi XL (2012). World J. Gastroenterol..

