# Supplementary material for: Hepatic spheroids derived from human induced pluripotent stem cells in bio-artificial liver rescue porcine acute liver failure
Source: Cell Res. 2019 Dec 11;30(1):95–7. doi: 10.1038/s41422-019-0261-5 (PMC6951340; doi:10.1038/s41422-019-0261-5)
Supplement: Supplementary file 1 — Supplementary information [file 41422_2019_261_MOESM1_ESM.pdf]

Supplementary information

Materials and Methods

hiPSC Culture and Differentiation

The present study was approved by the Clinical Research Ethics Committee of China-Japan Friendship Hospital (Ethical approval No. 2009-50) and the Stem Cell Research Oversight of Peking University (SCRO201103-03), and was conducted according to the principles of the Declaration of Helsinki.

Three undifferentiated hiPSC lines (C1, C3 & C5) were generated using an episomal reprogramming protocol (episomal vectors were obtained from Addgene). These hiPSC lines were maintained in E8 essential medium (Gibco, A15169) in a 37 °C incubator at 5% CO_2 and 100% humidity. For initiation of cell differentiation, hiPSCs were seeded at 5×10^5 cells per well of a 6-well plate in E8 essential medium containing 10 μM Y27632. By optimization of our previous differentiation protocol, we generate large-scale functional hepatocytes differentiated from hiPSCs as described below:

hiPSCs were induced into primitive streak with the combination of 100 ng/ml Activin A (Stemimmune), 0.5 ng/ml BMP4 (Stemimmune), 10 ng/ml bFGF (Peprotech) and 20 ng/ml Wnt3a for 1 day in RPMI1640 medium (Invitrogen) with B27 supplement (Invitrogen) and Pen Strep (Gibco), and were induced into definitive endoderm cells with the combination of 100 ng/ml Activin A, 0.5 ng/ml BMP4 and 10 ng/ml bFGF for 3 days. The hiPSC-derived endoderm cells were further specified into foregut endoderm cells with the combination of 20 ng/ml KGF (Stemimmune) and 5

μM SB431542 (Selleck) for 2 days in RPMI1640 medium with B27 supplement and Pen Strep. hiPSC-derived foregut endoderm cells were then induced to differentiate into hepatoblasts with the combination of 20 ng/ml KGF, 20 ng/ml BMP4, 10 ng/ml BMP2 (Stemimmune) and 10 ng/ml bFGF for another 3 days in RPMI1640 medium with B27 supplement and Pen Strep. At this stage, the hepatoblasts could be expanded in the optimized hHPC expansion medium: DMEM/F12 medium (Invitrogen) with B27 supplement and 10 μM forskolin (Selleck), 5 μM SB431542, 20 ng/ml EGF (Peprotech), 3 μM CHIR99021 (Selleck), 5 μM LPA (Santa Cruz), 1 μM Dex (Selleck) and 0.5 μM S1P (Santa Cruz). The hiPSC-derived hHPCs were induced to differentiate into mature hepatocytes with the hHPC maturation medium: Williams' E medium (Invitrogen) with B27 supplement, 50 μM forskolin and 10 μM SB431542.

Cryopreservation conditions of the hiPSC-derived hHPCs: 40% hHPC expansion medium, 50% FBS (PAN Biotech, P30-3302), 10% DMSO (Gibco) and 10 μM Y27632.

Fetal human hepatocytes (FHHs) were isolated from 14-week-old human embryos obtained from three abortion donors with informed patient consent. The fetal liver tissue was digested in DMEM/F12 medium supplemented with 1 mg/ml collagenase IV (Gibco) in a 37 °C incubator for 20 min, then dissociated to obtain single cells by repeated pipette action. The single cell suspension was centrifuged at 1000 rpm for 3 min, washed 3 times with DMEM/F12 medium, then plated on Matrigel (1:50 dilution) at 1×10^6 cells per well of a 12-well plate in hHPC expansion medium. The adherent cells were collected after 2-h culture for detection and analysis.

Primary human hepatocytes (PHHs) were isolated from leftover human donor livers resections. Liver tissue were perfused with collagenase IV until the tissue was no longer compact. Digested tissue was then separated with Adson forceps. The single cell suspension was washed 3 times with HCM (Lonza) and collected for detection and analysis.

HepG2 cells were cultured in DMEM supplemented with 10% FBS.

Bio-artificial Liver System

1. Large-Scale Expansion and Maturation

hHPC expansion was conducted with initial cell number of 1×10^7 cells, and expanded to $\sim 1 \times 10^9$ cells with continuous cell passage (Supplementary information, Fig. S5a). For cell passage, hHPCs were digested with Accutase (Merck) in a 37 °C incubator for 3-5 min, after which cells were collected and centrifuged at 1000 rpm for 3 min. Single cells were resuspended and plated at a split ratio of 1:2-1:3 onto cell culture dishes pre-coated with Matrigel (1:50 dilution).

After hHPCs were expanded to $\sim 1 \times 10^9$ cells, the hHPCs were transferred into a low-speed stirring floating culture system with 1 L hHPC expansion medium for ~3 days. The medium was then replaced with hHPC maturation medium and cultured for ~20 days to generate functional hepatic spheroids. The magnetic stirring apparatus was applied at 60 rpm for both these steps.

The functional hepatic spheroids were then transferred into cell bags and transported from Beijing to Nanjing, where they were cultured overnight. The hepatic

spheroids suspended in 500 ml hHPC maturation medium (without sera) were assembled into the substratum of the bioreactor. The assembled BAL device was maintained at 37 °C and 5% CO_2 in an incubator throughout the treatment duration.

2. Bio-artificial Liver System

Chinese Bama miniature pigs (~50 kg, either sex) were purchased from the Laboratory Animal Center of the Affiliated Drum Tower Hospital of Nanjing University Medical School. All animal procedures were performed according to institutional and national guidelines and approved by the Animal Care Ethics Committee of Nanjing University and Nanjing Drum Tower Hospital.

The hiPSC-derived hepatocyte BAL support system consisted of three circuits, two blood circuits and one cell circuit (Supplementary information, Fig. S7a). The whole system included three roller pumps, a heparin pump, an infusion heater, a plasma filter (Sorin Group Italia, Mirandola, Italy), a plasma component separator (Kawasumi Laboratories Inc, Tokyo, Japan), an oxygenation device, and a multilayer radial-flow bioreactor containing polystyrene nanofiber scaffolds. The incubator of the bioreactor and oxygenation device was maintained at 37 °C.

Pigs in all groups were intravenously injected with D-galactosamine (D-gal, Sigma, G0500) (0.4 g/kg) the day before BAL treatment (day 0), and the baseline blood sample was collected. Catheters were inserted into the internal jugular vein of the pigs under continuous anesthesia by intravenous administration of propofol (10 mg/kg/h) (Diprivan; AstraZeneca, Wuxi, China), and the catheters were then

connected to the BAL device. Whole blood of ALF pigs was channeled through the BAL devices for 4 h, which consisted of 3 circuits. Whole blood was perfused at a rate of 40 ml/min in the first blood circuit, following which plasma was separated via the plasma filter at a rate of 15 ml/min the second blood circuit. Finally, the plasma was channeled through the plasma component exchanger, counter current to the flow of the cell circuit for sufficient exchange, at a rate of 15 ml/min. After each BAL treatment, the culture medium was drained and hiPSC-derived hepatic spheroids were washed in hHPC maturation medium. Whole blood samples were collected at regular intervals (before treatment, 1-7 days after treatment) until completion of the study 7 days after treatment.

mRNA Expression Analysis

Total RNA was isolated using the Direct-zol RNA Miniprep Kit (ZYMO Research) and reverse-transcribed with TransScript First-Strand cDNA Synthesis SuperMix (TransGen Biotech). RT-qPCR was performed using KAPA SYBR® FAST Universal qPCR Mix (KAPA Biosystems) on the BIO-RAD CFX384TM Real-time System. Quantified values were normalized to the input determined by house-keeping genes (RRN18S). The RT-qPCR primer sequences are provided in Supplementary information, Table S6.

Flow Cytometry

Differentiated cells were dispersed into single-cell suspensions by digestion in

Accutase at 37 °C for 3-5 min and then washed in PBS. The cell suspension was fixed in fixation/permeabilization solution buffer (BD, 554714) at 4 °C for 20 min and washed 3 times with perm/wash buffer (BD, 554723). The cells were then resuspended in perm/wash buffer with primary antibodies and incubated at 4 °C overnight. Cells were then washed 3 times with wash buffer and incubated in perm/wash buffer with secondary antibodies at for 1-2 h at 4 °C. The cells were then washed three times and analyzed using a flow cytometer (Beckman CytoFlex).

Human Albumin and Urea Detection

Human albumin was measured using the Human Albumin ELISA Quantitation kit (Bethyl Laboratory, E80-129) according to the manufacturer's instructions. Urea synthesis was measured using the QuantiChrom Urea Assay Kit (BioAssay System, BA_DIUR-500) according to the manufacturer's instructions.

Ammonia Elimination Detection

To detect the ammonia elimination abilities, cells were incubated in hHPC maturation medium supplemented with 200 μM NH_4Cl for 24 h. $NH4^+$ concentrations were detected by Ortho Clinical Diagnostics (FS5600, Johnson and Johnson Company).

PAS Staining

The PAS staining system was purchased from Sigma-Aldrich. Cultures were fixed with 4% paraformaldehyde (DingGuo) and stained according to the manufacturer's

instructions.

Immunofluorescence

Cultured cells were washed with PBS and fixed in 4% paraformaldehyde at room temperature for 15 min. They were then washed 3 times with PBS for 3 min and then blocked with PBS containing 0.25% Triton X-100 and 5% normal donkey serum (Jackson ImmuneResearch Laboratories, Inc) at room temperature for 1 h or at 4 °C overnight. The samples were incubated with primary antibodies at 4 °C overnight, washed three times with PBS, and then incubated with the appropriate secondary antibodies for 1 h at room temperature in the dark. Nuclei were stained with DAPI (Roche). The primary and secondary antibodies used for immunostaining are listed in Supplementary information, Table S7.

Growth curve and doubling times

To calculate the doubling time of hHPCs in the expansion stage, cells were plated at a density of 5×10^5 cells per well in hHPC expansion medium and cultured in a 12-well plate coated with Matrigel (1:50). The growth rate was determined by counting the cells number using a hemocytometer as a function of time. Data from the exponential phase of growth (data points at each passage time, passage ratio 1:2 to 1:3) were used to generate an exponential growth curve.

CYP Metabolism Assay: LC-MS

The hMHs and PHHs were dissociated and suspended to measure CYP450 activities. The cell suspension was diluted to 1×10^6 cells/ml in reaction buffer, which consisted of William's E medium (Gibco) with Glutamax (Gibco, 100×) and HEPES (Gibco, 100×). The cell mixture was 1:1 resuspended in a 2× concentration of substrate buffer in a 500 μl reaction system. After 15-min incubation at 37 °C in an orbital shaker (~200 rpm/min), the reactions were stopped by addition of sample aliquots to the tubes containing triple the volume of quenching solvent (methanol) and frozen at -80 °C. Isotope-labeled reference metabolites were used as internal standards for further LC-MS analysis. The metabolites were quantified using a Triple Quad 4500 using validated traditional LC-MS methods.

RNA-Seq analysis

Total RNA of hiPSCs, hHPCs, hMHs, FHHs and PHHs were isolated using the RNeasy Micro Kit (Qiagen) and then sent to a private sequencing company for detection and analysis. RNA sequencing libraries were prepared with the IlluminaTruSeq RNA Sample Preparation Kit. The fragmented and randomly primed 200-bp paired-end libraries were sequenced on the IlluminaHiSeq 4000 sequencing system.

Statistics

For most statistic evaluation, an unpaired *t*-test was applied to calculate statistical probability in this study. *P* values were calculated by two-tailed test.

Supplementary information, Figure S1

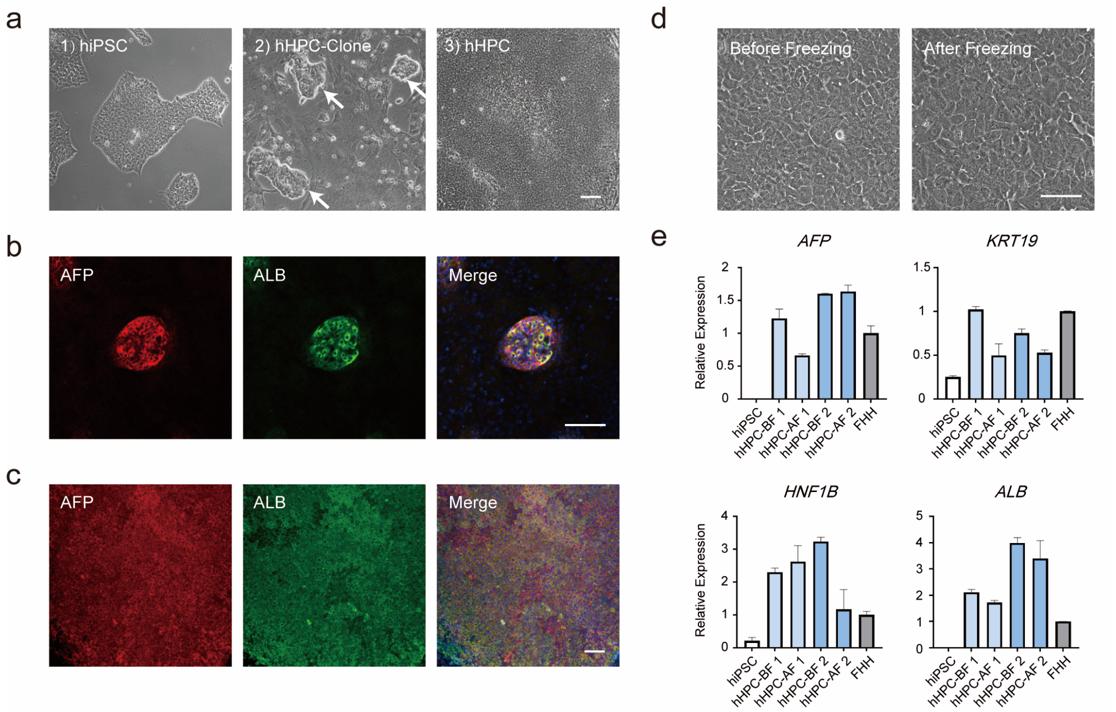

Fig. S1 **a** Cell morphology of hiPSCs differentiated into hHPCs. 1) Undifferentiated hiPSCs; 2) hHPCs in hHPC expansion medium. White arrow: hepatic progenitor colonies; 3) Expansion of hHPCs in hHPC expansion medium at passage 0. Scale bar, 100 μm. **b** Immunofluorescence analysis of hHPC clones express the hepatic progenitor markers AFP (α-fetoprotein) and ALB (albumin). Scale bar, 100 μm. **c** Immunofluorescence analysis of expanded hHPCs at passage 0 in hHPC expansion medium express the hepatic progenitor markers AFP and ALB. Scale bar, 100 μm. **d** Cell morphology of the hHPCs before cell freezing or after cell recovery. Scale bar, 100 μm. **e** Quantitative comparison of the expression of hepatic progenitor specific genes in hiPSC-derived hHPCs before or after cell freezing (BF or AF). hiPSCs and FHHs served as controls. n = 2.

Supplementary information, Figure S2

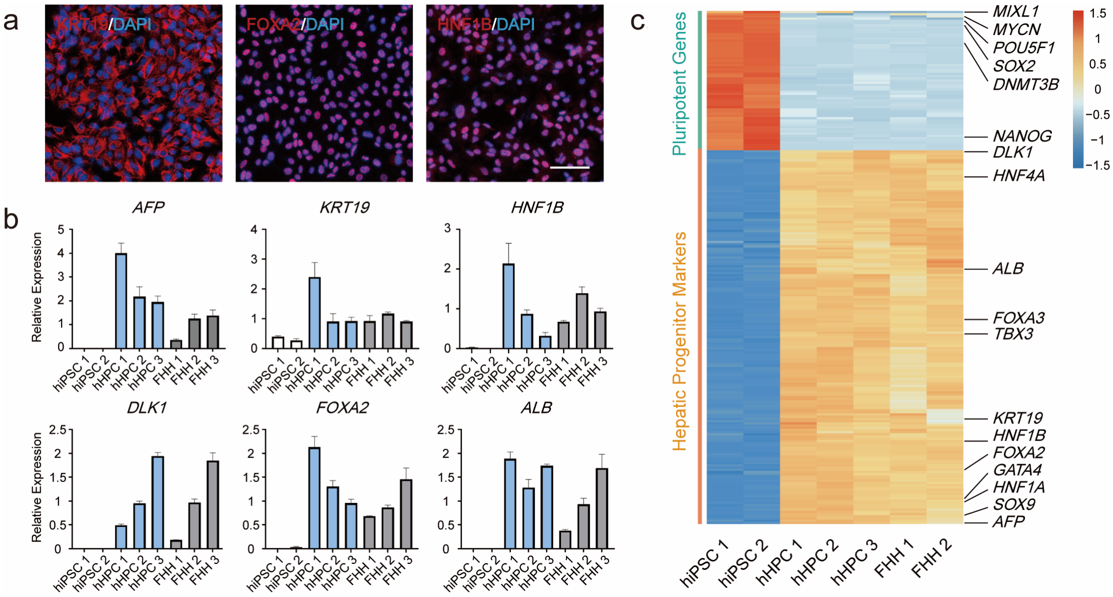

Fig. S2 a Immunofluorescence analysis of the expression of hepatic progenitor-specific markers KRT19, FOXA2 and HNF1B in hHPCs. Scale bar, 100 μm. **b** Quantitative comparison of the hepatic progenitor specific gene expression levels among hiPSCs, hHPCs and FHHs. hHPCs 1-3 represent hHPCs differentiated from three individual hiPSC lines. n = 4. Data are presented as means ± SEM. *t*-test, $P > 0.05$. **c** Heatmap of pluripotent gene and hepatic progenitor marker gene expression profiles of hiPSCs, hHPCs and FHHs by RNA-Seq analysis.

Supplementary information, Figure S3

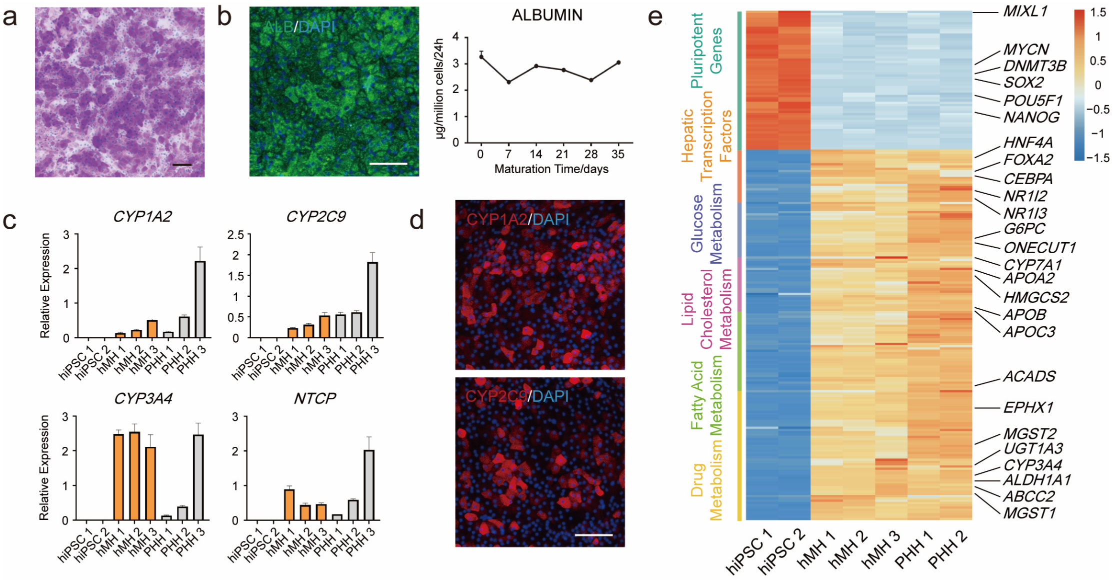

Fig. S3 **a** Glycogen synthesis in hMHs, as detected by Periodic Acid-Schiff (PAS) staining. Scale bar, 100 µm. **b** Immunofluorescence analysis and ELISA detection of the expression of albumin. The expression level of albumin remained stable during the hepatic maturation. Scale bar, 100 µm. **c** Quantitative comparison of gene expression levels of drug metabolism-related genes among hiPSCs, hMHs and PHHs. hMHs 1-3 represent hMHs differentiated from three individual hiPSC lines. n = 4. Data are presented as means ± SEM. *t*-test, $P > 0.05$. **d** Immunofluorescence analysis of the expression of the drug metabolism-related genes CYP1A2 and CYP2C9. Scale bar, 100 µm. **e** Heatmap comparing the gene expression profiles of multiple batches of hiPSCs, hMHs and PHHs in terms of pluripotent genes and hepatic functions of drug metabolism, fatty acid metabolism, lipid cholesterol metabolism, glucose metabolism and hepatic transcription factors by RNA-Seq analysis.

Supplementary information, Figure S4

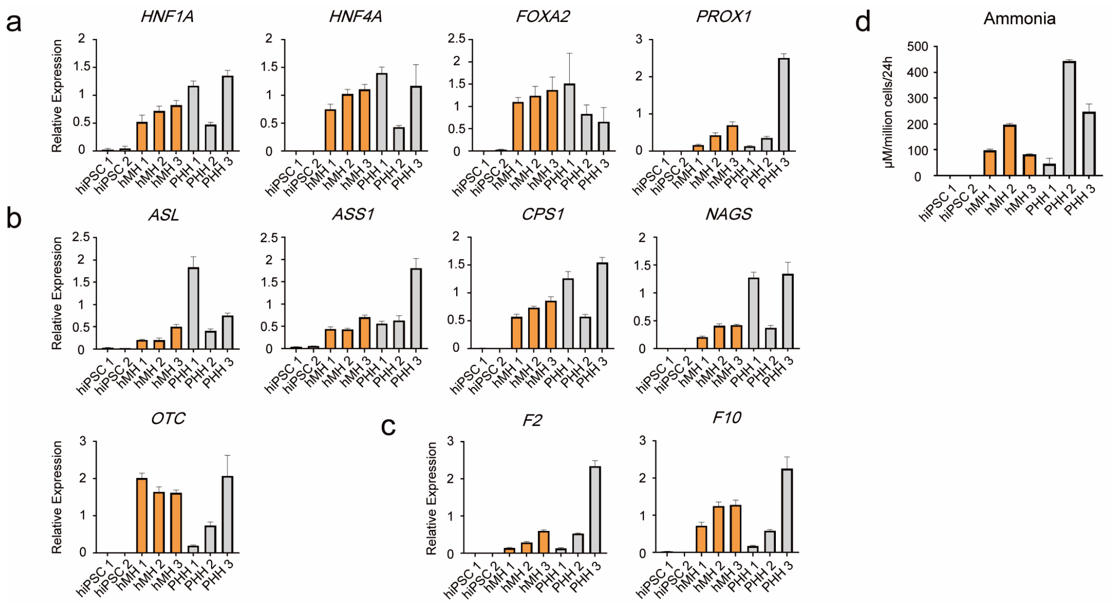

Fig. S4 a-c Quantitative comparison of gene expression levels of mature hepatocyte transcription factors (**a**), urea synthesis-related genes (**b**) and blood coagulation factors (**c**) among hiPSCs, hMHs and PHHs. hMHs 1-3 represent hMHs differentiated from three individual hiPSC lines. n = 4. Data are presented as means ± SEM. *t*-test, $P > 0.05$. **d** Quantitative comparison of ammonia elimination levels among hiPSCs, hMHs and PHHs. hMHs 1-3 represent hMHs differentiated from three individual hiPSC lines. n = 2. Data are presented as means ± SEM. *t*-test, $P > 0.05$.

Supplementary information, Figure S5

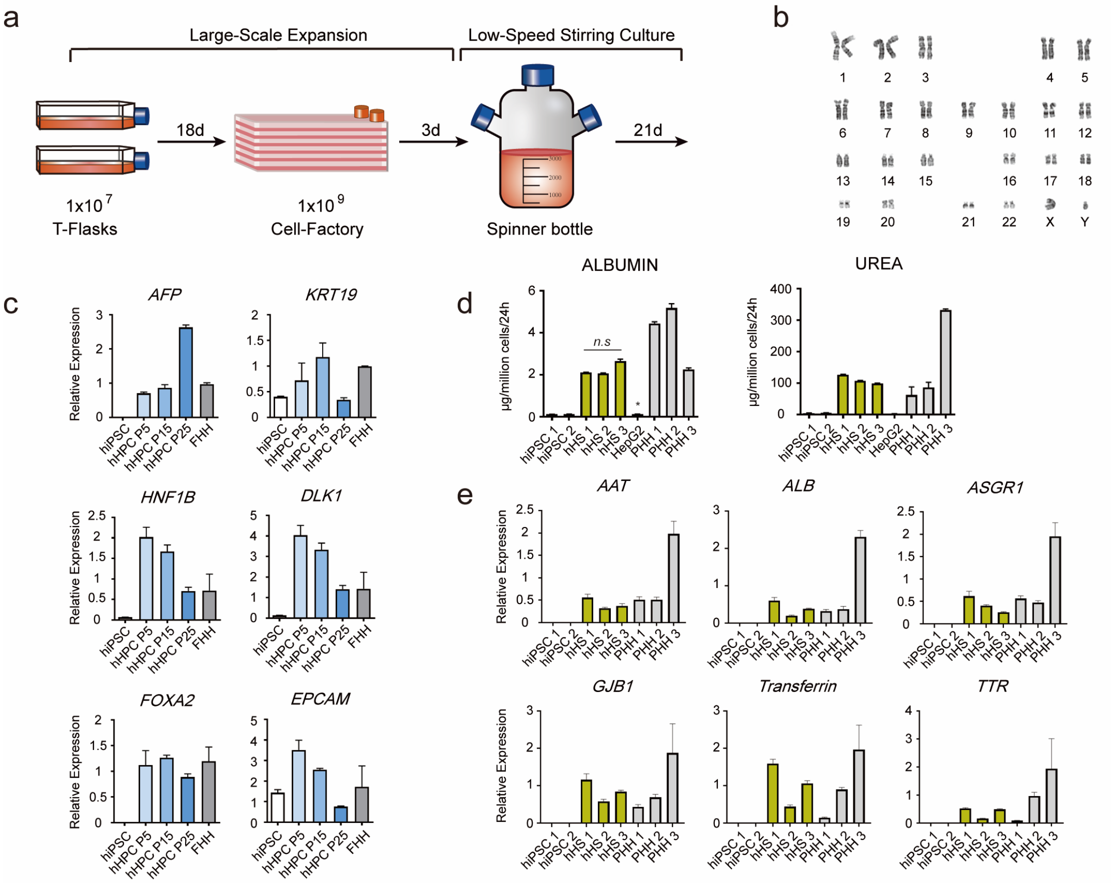

Fig. S5 a Schematic of the large-scale expansion and maturation of hiPSC-derived hHPCs. The large-scale expansion of hHPCs was initiated with 1 × 10^7 cells and expanded to 1 × 10^9 cells in ~20 days by continuous cell passage. The hHPCs were then transferred to a low-speed stirring culture system for floating culture for ~20 days to generate the functional hepatic spheroids. **b** Karyotype detection of hiPSC-derived hHPCs at passage 25. **c** Quantitative comparison of the expression of hepatic progenitor-specific genes among hiPSCs, hHPCs (P5, passage 5; P15, passage 15; P25, passage 25) and FHHs. n = 2. Data are presented as means ± SEM. **d** Quantitative analysis of albumin secretion and urea synthesis among hiPSCs, hHSs,

HepG2 cells and PHHs. hHSs 1-3 represent hHSs differentiated from three individual hiPSC lines. n = 2. Data are presented as means ± SEM. *t*-test, $*P < 0.05$. **e** Quantitative comparison of the expression of clinically related basic hepatic markers among hiPSCs, hHSs and PHHs. hHSs 1-3 represent hHSs differentiated from three individual hiPSC lines. n = 4. Data are presented as means ± SEM. *t*-test, $P > 0.05$.

Supplementary information, Figure S6

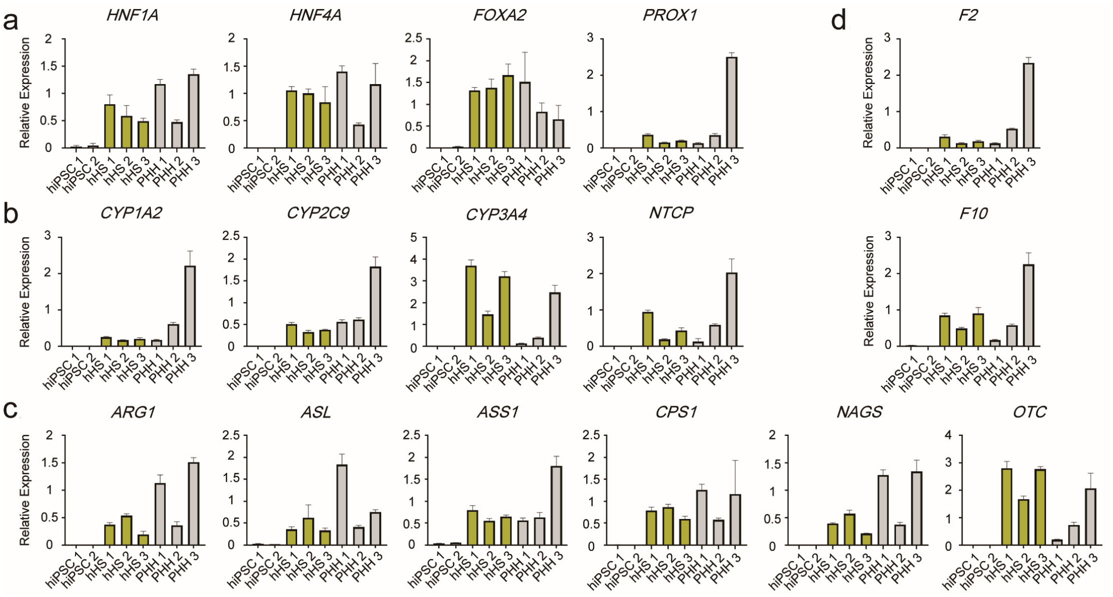

Fig. S6 a-d Quantitative comparison of gene expression levels of mature hepatocyte transcription factors (**a**), drug metabolism-related genes (**b**), urea synthesis-related genes (**c**) and blood coagulation factors (**d**) among hiPSCs, hHSs and PHHs. hHSs 1-3 represent hHSs differentiated from three individual hiPSC lines. n = 4. Data are presented as means ± SEM. *t*-test, $P > 0.05$.

Supplementary information, Figure S7

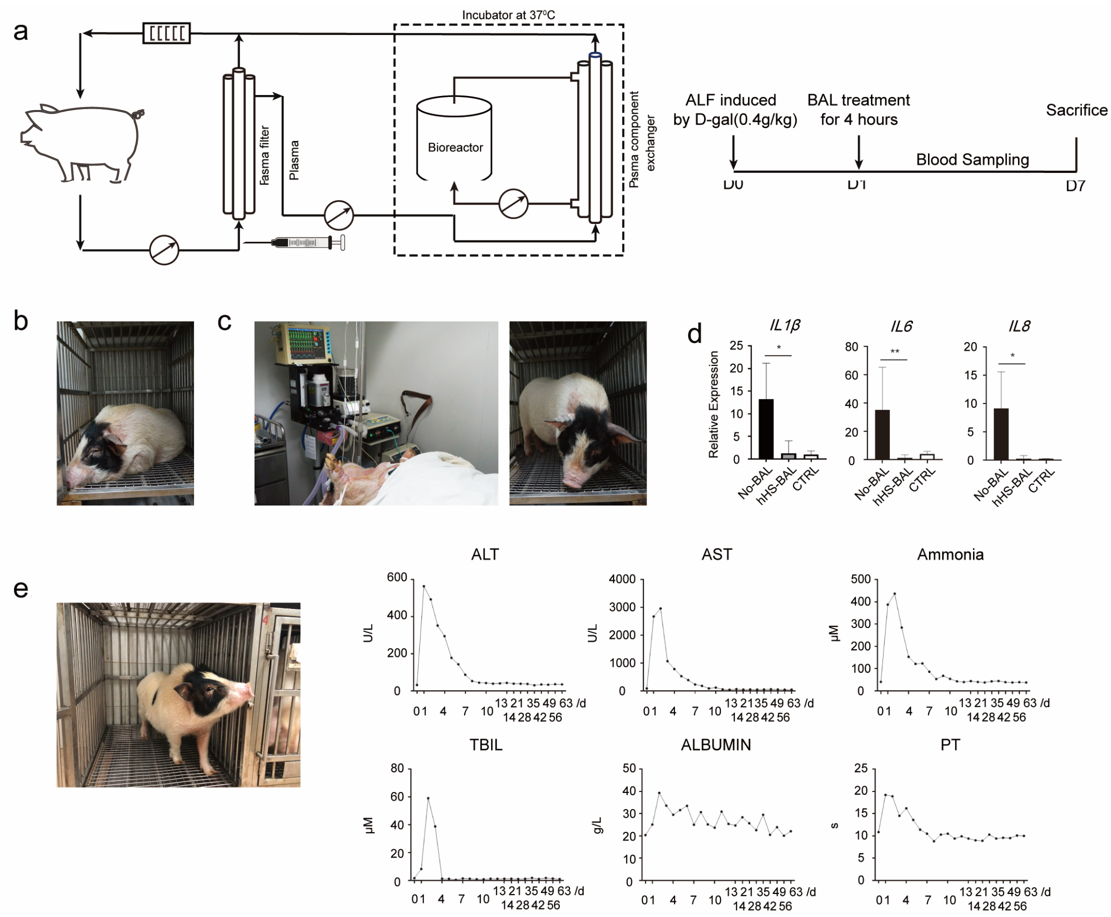

Fig. S7 **a** Schematic view of the hiPSC-derived hepatic spheroid supported BAL system. Approximately 1 × 10^9 cells were suspended in the bioreactor during the BAL treatment. The ALF pigs were induced with D-gal on Day 0; and treated with BAL system on Day 1 for 4 h. The blood samples of ALF pigs were collected every day until day 7. **b** Liver failure state of the ALF pigs before BAL treatment. **c** The ALF pig treated with hHS-BAL system (left) and the state of the hHS-BAL-treated ALF pig on Day 2 after ALF induction (right). **d** Quantitative comparison of expression of inflammatory cytokine genes in the No-BAL ALF group and hHS-BAL treatment ALF group. Healthy porcine liver sample served as negative control. Samples were

selected randomly to represent the No-BAL ALF group (n = 2), the hHS-BAL treatment ALF group (n = 6) and the healthy porcine liver samples (n = 2). Data are presented as means ± SEM. *t*-test, **P* < 0.05, ***P* < 0.01. **e** Long-term observation of an additional hHS-BAL-treated ALF pig. Two months after BAL treatment, this pig showed excellent survival, without signs of ALF symptoms. The blood physiological indices of this pig including ALT, AST, ammonia and TBIL levels were restored to healthy levels in 7 days, and they were subsequently maintained.

Supplementary information, Table S1

Information of ALF pigs

Summary of experimental groups: Weight and survival times of ALF pigs as well as total cell number contained in their respective BAL devices in the hHS-BAL treatment group (**A**), non-treated group (**B**) or empty-BAL treated group (**C**).

A. hHS-BAL treatment group

Group	Pig weight (kg)	Cell number	Survival
1	48.5	1.0×10^9	Survived
2	47.3	1.1×10^9	Survived
3	53.1	0.8×10^9	Survived
4	49.4	1.2×10^9	Survived
5	48.7	1.0×10^9	Survived
6	52.5	1.2×10^9	Survived

All survived animals were sacrificed on day 7.

B. No-BAL treatment group

Group	Pig weight (kg)	Cell number	Survival
1	51.5	--	2 days
2	49.1	--	4 days
3	53.3	--	2 days
4	50.1	--	3 days
5	49.8	--	3 days
6	48.3	--	2 days

C. Empty-BAL treatment group

Group	Pig weight (kg)	Cell number	Survival
1	53.0	--	2 days
2	52.3	--	2 days
3	47.6	--	3 days
4	49.9	--	3 days
5	50.8	--	3 days
6	51.5	--	4 days

Supplementary information, Table S2

Detection of serum biochemical indices in hHS-BAL group.

ALT (U/L)								
Day	0	1	2	3	4	5	6	7
1	46.6	415.5	313.0	328.5	348.1	277.8	205.9	137.5
2	28.7	262.0	112.6	87.8	73.6	61.2	60.2	46.9
3	54.5	329.2	182.4	89.5	77.2	69.9	55.0	44.4
4	47.6	379.3	145.4	127.4	90.4	60.5	50.4	44.7
5	44.5	301.3	179.4	109.5	79.2	89.9	65.2	45.2
6	42.1	452.3	363.4	182.1	78.3	72.7	55.1	39.3

AST (U/L)								
Day	0	1	2	3	4	5	6	7
1	102.0	3109.2	966.4	867.4	968.3	943.3	771.9	359.5
2	15.5	1879.6	156.8	52.6	35.3	29.0	75.3	19.6
3	42.5	932.3	156.7	82.1	86.7	79.0	61.3	54.5
4	39.8	1824.9	547.4	254.7	77.4	55.4	44.9	33.4
5	46.3	892.4	144.2	99.3	76.4	77.3	56.3	50.2
6	47.0	1312.4	144.8	113.4	83.4	68.9	53.4	47.5

Ammonia (μM)								
Day	0	1	2	3	4	5	6	7
1	68.1	330.4	216.3	161.3	170.5	156.4	183.2	123.3
2	10.5	253.7	35.1	26.6	78.3	33.1	69.5	29.1
3	45.4	229.2	243.6	102.5	112.3	50.8	53.4	41.2
4	24.4	325.2	45.5	28.3	33.4	26.9	14.7	15.6
5	55.2	283.8	223.4	124.6	121.3	77.3	54.1	46.9
6	55.7	199.1	121.8	76.3	65.1	54.3	51.6	55.1

TBIL (μM)								
Day	0	1	2	3	4	5	6	7
1	5.4	163.2	116.4	63.5	28.9	24.2	30.5	1.5
2	0.8	34.2	26.0	2.6	1.2	1.3	12.0	1.2
3	0.3	0.9	1.3	1.0	0.8	1.4	1.2	1.1
4	0.2	4.2	1.1	0.9	1.1	0.9	0.7	0.9
5	0.4	1.0	1.5	1.4	1.3	1.2	1.3	1.0
6	0.3	2.3	1.3	1.1	1.2	1.2	0.9	0.8

Albumin (g/L)								
Day	0	1	2	3	4	5	6	7
1	30.7	42.4	41.4	39.3	46.0	41.6	43.5	55.0
2	42.0	45.5	35.4	35.7	37.5	37.8	38.9	37.7
3	41.0	44.0	29.4	42.5	36.4	29.9	37.0	31.3
4	39.2	35.1	35.1	39.6	35.7	34.3	36.8	37.4
5	44.0	45.1	30.0	35.0	36.0	33.0	32.0	32.0
6	38.3	39.4	32.7	30.8	28.4	27.4	30.3	30.1

PT (s)								
Day	0	1	2	3	4	5	6	7
1	8.1	19.3	59.9	27.4	11.1	13.4	8.2	8.2
2	8.5	22.0	11.0	9.0	8.1	7.1	7.5	7.1
3	29.4	28.4	18.4	29.4	29.4	27.8	21.8	22.4
4	10.5	29.3	14.5	17.3	10.4	9.9	8.7	8.6
5	11.0	25.4	18.4	15.6	14.4	14.0	12.1	11.1
6	10.8	22.8	18.9	17.0	16.2	14.4	13.4	10.1

Supplementary information, Table S3

Detection of serum biochemical indices in No-BAL group.

ALT (U/L)								
Day	0	1	2	3	4	5	6	7
1	32.5	442.3						
2	28.3	302.5	506.1	692.4				
3	26.3	491.6						
4	23.3	557.7	639.3					
5	23.0	358.2	463.8					
6	39.6	383.6						

AST (U/L)								
Day	0	1	2	3	4	5	6	7
1	44.7	2082.4						
2	46.3	1156.5	2458.4	3742.5				
3	32.5	1624.8						
4	44.9	1684.3	2745.7					
5	34.4	1458.5	2493.5					
6	44.7	3435.9						

Ammonia (µM)								
Day	0	1	2	3	4	5	6	7
1	30.3	319.7						
2	24.2	205.7	429.3	533.4				
3	10.2	343.8						
4	19.3	225.2	583.7					
5	20.2	125.4	498.4					
6	20.0	483.2						

TBIL (µM)								
Day	0	1	2	3	4	5	6	7
1	8.2	25.9						
2	10.8	22.9	60.5	52.7				
3	8.2	28.4						
4	9.9	59.5	72.9					
5	8.0	24.1	35.3					
6	8.1	45.2						

Albumin (g/L)								
Day	0	1	2	3	4	5	6	7
1	40.2	36.2						
2	43.3	39.7	37.5	39.2				
3	32.7	40.1						
4	31.0	38.3	40.0					
5	39.5	38.7	43.0					
6	41.6	30.0						

PT (s)								
Day	0	1	2	3	4	5	6	7
1	9.4	28.5						
2	10.3	20.8	25.4	33.5				
3	10.6	29.5						
4	9.9	30.6	34.2					
5	9.2	25.2	29.8					
6	10.2	32.4						

Supplementary information, Table S4

Detection of serum biochemical indices in Empty-BAL group.

ALT (U/L)								
Day	0	1	2	3	4	5	6	7
1	65.4	583.7						
2	21.2	297.8						
3	31.4	364.7	689.7					
4	44.3	451.0	582.6					
5	23.9	252.6	434.9					
6	44.8	326.8	482.7	644.7				

AST (U/L)								
Day	0	1	2	3	4	5	6	7
1	72.7	2983.8						
2	59.8	3201.8						
3	42.3	1741.2	2576.8					
4	50.0	1384.5	3051.9					
5	45.5	1954.2	2546.4					
6	60.6	3076.5	3689.5	4520.7				

Ammonia (μM)								
Day	0	1	2	3	4	5	6	7
1	20.2	523.2						
2	39.7	445.3						
3	20.4	201.3	578.3					
4	20.9	301.5	513.5					
5	28.6	209.9	414.8					
6	9.3	367.3	461.0	694.1				

TBIL (μM)								
Day	0	1	2	3	4	5	6	7
1	12.9	49.0						
2	18.1	21.6						
3	9.4	38.3	65.1					
4	9.7	51.0	77.0					
5	9.2	33.0	46.5					
6	7.2	19.1	49.8	59.0				

Albumin (g/L)								
Day	0	1	2	3	4	5	6	7
1	51.3	28.5						
2	32.6	29.2						
3	22.6	29.6	27.8					
4	39.2	33.5	28.8					
5	33.1	30.7	28.6					
6	40.6	34.3	38.1	27.4				

PT (s)								
Day	0	1	2	3	4	5	6	7
1	9.1	34.0						
2	12.5	31.0						
3	9.6	26.8	30.5					
4	11.0	28.8	33.0					
5	9.3	26.8	29.3					
6	10.5	25.2	29.8	31.9				

Supplementary information, Table S5

Detection of serum biochemical indices of the hHS-BAL-treated ALF pig for long-term observation.

Day	0	1	2	3	4	5	6	7
ALT (U/L)	32.1	563.6	493.1	351.5	295.1	178.3	143.9	87.3
AST (U/L)	88.6	2671.4	2962.4	1069.3	789.5	531.9	393.2	232.5
Ammonia (μM)	40.5	387.4	436.3	284.5	153.2	121.4	123.5	86.3
TBIL (μM)	1.7	8.3	59.1	38.8	1.3	1.2	0.5	1.5
Albumin (g/L)	20.4	25.1	39.3	33.6	29.5	31.6	33.5	25.0
PT (s)	10.9	19.2	18.9	14.5	16.2	13.6	11.4	10.5

Day	8	9	10	11	12	13	14	21
ALT (U/L)	53.1	44.5	42.7	39.4	40.6	43.5	39.3	38.5
AST (U/L)	181.2	92.3	122.1	57.4	45.2	63.9	52.6	55.7
Ammonia (μM)	53.6	68.3	54.2	43.1	39.8	44.5	40.2	38.3
TBIL (μM)	1.3	0.9	0.9	1.3	1.2	1.3	1.3	1.1
Albumin (g/L)	30.7	25.2	23.7	30.9	25.4	24.7	28.4	25.7
PT (s)	8.8	10.3	10.5	9.4	9.9	9.4	9.0	8.9

Day	28	35	42	49	56	63
ALT (U/L)	37.8	30.5	34.6	33.8	36.1	35.2
AST (U/L)	50.6	56.4	60.9	53.4	49.3	48.9
Ammonia (μM)	43.1	44.6	39.4	38.1	39.2	38.3
TBIL (μM)	1.2	1.9	1.1	1.8	1.4	1.0
Albumin (g/L)	22.6	29.5	20.5	23.9	20.1	22.1
PT (s)	10.3	9.4	9.6	9.5	10.1	10.0

Supplementary information, Table S6

Primers used for mRNA expression detection.

No.	Gene Name		Primers
1	AAT	F	TATGATGAAGCGTTTAGGC
		R	CAGTAATGGACAGTTTGGGT
2	AFP	F	CCCGAACTTTCCAAGCCATA
		R	TACATGGGCCACATCCAGG
3	ALB	F	GCACAGAATCCTTGGTGAACAG
		R	ATGGAAGGTGAATGTTTCAGCA
4	ARG1	F	GTGGAAACTTGCATGGACAAC
		R	AATCCTGGCACATCGGGAATC
5	ASGR1	F	ATGACCAAGGAGTATCAAGACCT
		R	TGAAGTTGCTGAACGTCTCTCT
6	ASL	F	CAGTGGACCCCATCATGGAGA
		R	GGCTTTGCTGCCTTGAACATC
7	ASS1	F	CTTGGGGCCAAAAAGGTGTTC
		R	GAGGTAGCGGTCCTCATACAG
8	CEBPA	F	ACAAGAACAGCAACGAGTACCG
		R	CATTGTCACTGGTCAGCTCCA
9	CPS1	F	AATGAGGTGGGCTTAAAGCAAG
		R	AGTTCCACTCCACAGTTCAGA
10	CYP1A2	F	CTTCGTAAACCAGTGGCAGG
		R	AGGGCTTGTTAATGGCAGTG
11	CYP2C9	F	GCCACATGCCCTACACAGATG
		R	TAATGTCACAGGTCACTGCATGG
12	CYP2D6	F	GTGTCCAACAGGAGATCGACG
		R	CACCTCATGAATCACGGCAGT
13	CYP3A4	F	GGTGGTGAATGAAACGCTCAG
		R	ACCCCTTTGGGAATGAACATC
14	DLK1	F	GGGCACAGGAGCATTCATAG
		R	GACGGGGAGCTCTGTGATAG
15	EPCAM	F	AGGAGATGGGTGAGATGC
		R	GATTGGTAAAGCCAGTTTC
16	F10	F	CACTGGTCGCCATCTTTGTA
		R	AGTGCATGGAAGAGACCTGC
17	F2	F	GGCTCTTCATGACAAAGGGT

		R	ATCCGCATCACTGACAACAT
18	FOXA1	F	GTGGCTCCAGGATGTTAGGA
		R	AGGCCTGAGTTCATGTTGCT
19	FOXA2	F	CGACTGGAGCAGCTACTATGC
		R	TACGTGTTCATGCCGTTCAT
20	GATA4	F	CCCGACACCCCAATCTC
		R	CAGGCGTTGCACAGATAGTG
21	GJB1	F	ATGCTCCGACAGCGTCTC
		R	TGCCCTCTGCTCCTCTTAC
22	HHEX	F	ACGGTGAACGACTACACGC
		R	CGTTGGAGAATCTCACCTGG
23	HNF1A	F	CCATCCTCAAAGAGCTGGAG
		R	GTGCTGCTGCAGGTAGGACT
24	HNF1B	F	GCACCTCTCCCAGCATCTCA
		R	GTCGGAGGATCTCTCGTTGC
25	HNF4A	F	ACTACATCAACGACCGCCAGT
		R	ATCTGCTCGATCATCTGCCAG
26	IL1β	F	TCAGGCAGATGGTGTC
		R	CTGGGAGGAGGGATTC
27	IL6	F	AGAAGATGCCAAAGGT
		R	CGGAGAGGTGAAGAGC
28	IL8	F	CTCTCTGTGAGGCTGC
		R	TGGAAAGGTGTGGAAT
29	KRT19	F	TGAGCAGGTCCGAGGTTACTG
		R	CAGTGTGTCTTCCAAGGCAGC
30	NAGS	F	GTTGGAGAAGCTGCCATCAC
		R	CGGGACCTTCAGACACTTTT
31	NTCP	F	AGGGGGACATGAACCTCAG
		R	AGGTCCCCATCATAGATCCC
32	OTC	F	CGGCCCGTGTATTGTCTAGC
		R	TAGCCAGGGTGTCCAAATCTG
33	PROX1	F	ACAGGGCTCTGAACATGCAC
		R	GGCATTGAAAAACTCCCGTA
34	SOX9	F	GTAATCCGGGTGGTCCTTCT
		R	GACGCTGGGCAAGCTCT
35	Transferrin	F	TGTCTACATAGCGGGCAAGT
		R	GTTCCAGCCAGCGGTTCT

36	TTR	F	TGGGAGCCATTTGCCTCTG
		R	AGCCGTGGTGGAATAGGAGTA
37	UGT1A3	F	GCCAACAGGAAGCCACTATC
		R	CAGCAATTGCCATAGCTTTC

Supplementary information, Table S7

Antibodies used for immunofluorescence analysis or flow cytometry analysis.

No.	Protein Name	Dilution	Company
1	AFP	1:200	Invitrogen
2	ALB	1:200	Bethy
3	CYP1A2	1:1000	AbDSerotec
4	CYP2C9	1:1000	AbDSerotec
5	CYP2D6	1:1000	AbDSerotec
6	CYP3A4	1:1000	AbDSerotec
7	FOXA2	1:200	Abcam
8	HNF1B	1:200	Santa Cruze
9	Ki67	1:200	Abcam
10	KRT19	1:200	ZhongShan
11	Donkey anti-Mouse IgG, 550	1:200	Abcam
12	Donkey anti-Rabbit IgG, 550	1:200	Abcam
13	Donkey anti-Goat IgG, 488	1:200	Abcam
14	Donkey anti-Mouse IgG, 647	1:200	Abcam
